# Dynamic Mechanical Behavior Analysis of Flax/Jute Fiber-Reinforced Composites under Salt-Fog Spray Environment

**DOI:** 10.3390/polym12030716

**Published:** 2020-03-24

**Authors:** Vincenzo Fiore, Carmelo Sanfilippo, Luigi Calabrese

**Affiliations:** 1 Department of Engineering, University of Palermo, Viale delle Scienze, Edificio 6, 90128 Palermo, Italy; carmelo.sanfilippo01@unipa.it; 2 Department of Engineering, University of Messina, Contrada Di Dio (Sant’Agata), 98166 Messina, Italy; lcalabrese@unime.it

**Keywords:** damping, green composites, salt-fog exposition, aging behavior, surface treatment

## Abstract

Over the last decades, natural fiber-reinforced polymer composites (NFRPs) gained great attention in several engineering fields thanks to the reduction of the environmental impact and the end-of-life cost disposal. Unfortunately, the use of NFRPs is limited, mainly due to their weak resistance against humid environments. Since limited literature is available about the evolution of the dynamic mechanical response of NFRPs under aggressive environments, this paper aims to investigate the damping properties of flax, jute and flax/jute epoxy composites exposed to salt-fog up to 60 days. Furthermore, sodium bicarbonate fiber treatment was performed to improve the composites’ durability. The effectiveness of treatment was evidenced for full flax-reinforced composites, whereas no beneficial effect was found for jute composites. Moreover, treated hybrid laminates having outer laminae reinforced with flax showed better damping behavior than their hybrid counterparts during the whole aging campaign.

## 1. Introduction

Among natural fibers, flax [1] and jute [2] are nowadays widely utilized instead of synthetic counterparts such as glass in fiber-reinforced polymers (FRPs). Thanks to their specific mechanical properties [3] and good insulating features [4], flax and jute fibers have received great attention as reinforcements of composite components useful in several engineering applications [5,6,7,8,9]. 

Natural fiber-reinforced composites (NFRPs) have received growing attention in the last decades, both from the academic world and from industries, thanks to their specific mechanical properties, reduced cost and advantages for health and recyclability. Nevertheless, this class of innovative materials shows also some drawbacks, such as their limited aging resistance in humid or wet environmental conditions, i.e., an extensive literature shows that NFRP materials experienced dramatic degradation of their quasi-static mechanical properties when exposed to these environments [10,11,12,13,14,15,16,17,18]. This can be partially ascribed to the softening and plasticization phenomena of natural fibers and thermoset matrix [19,20]. In particular, different combined chemo-physical phenomena (i.e., plasticizing, swelling, and hydrolysis) occur in organic resins due to hydrothermal aging [21,22,23]. Furthermore, it is widely known that natural fibers show a high tendency to moisture absorption [24,25] due to the hydrophilic nature of their polysaccharide constituents (i.e., mainly cellulose and hemicellulose). In more detail, hemicellulose and the amorphous fraction of cellulose absorb water (due to the high percentage of hydroxyl groups), thus leading to a decrease of the tensile stability of natural fibers that become more flexible as a result of the plasticization effect [20,26]. 

In addition, the degradation phenomena of natural fibers are accelerated in seawater due to the presence of salts such as NaCl [27]. This latter is dissolved in the form of Na^+^ cations and Cl^−^ anions, which, being able to spread within the composite structure, favor local damages especially in its interstitial areas. This mechanism also stimulates an osmotic water diffusion at the fiber/matrix interface, which further speeds up the interfacial debonding phenomena [28,29]. 

Another issue that contributes to a great extent to the low aging resistance of NFRPs under humid environmental conditions is the poor compatibility between hydrophilic natural fibers and several hydrophobic polymeric matrices such as epoxy resins [30,31]. In this regard, some researchers showed that the water resistance of natural fiber-reinforced polymer composites can be improved and their quasi-static mechanical properties under humid environment can be better retained through fiber chemical treatments [32,33,34,35].

Despite the wide literature about the quasi-static properties, research concerning the evolution of the dynamic mechanical behavior of NFRPs under such critical environmental conditions [36,37,38,39] and mainly how fiber surface treatments can help these materials to retain their damping properties [40,41] is still limited to date. 

It is widely known that the knowledge of the damping properties such as storage modulus (E’), loss modulus (E’’), and loss factor (tan δ) provides essential information about the interfacial bonding between reinforcing fibers and the polymer matrix of composite materials [42]. For this reason, dynamic mechanical thermal analysis (DMTA) represents an indispensable and effective tool for analyzing the behavior of NFRPs under aggressive environments. Moreover, this characterization can furnish the designer with important data about the effectiveness of fiber treatments. Within this context, the beneficial effect of surface treatments such as alkalization, silanization, acetylation and alkali-silanization on the dynamic mechanical behavior of flax fiber-reinforced polymers (FFRPs) under hydrothermal aging was shown by Wang and Petru [40]. The water resistance and damping properties of FFRPs were improved after all surface treatments, even if the acetylation treatment led the best damping performance among all treated composites. Analogously, Doan et al. [41] showed that the dynamic mechanical behavior of jute/polypropylene composites under humid environmental conditions (i.e., 20 °C; 95% RH) can be improved by adding to the polymeric matrix an appropriate modifier (i.e., maleic anhydride-grafted polypropylene). 

Despite their beneficial effects on the fiber–matrix adhesion and, consequently, on the static and dynamic mechanical properties of NFRP materials, it is worth noting that the chemical methods performed in the past are considered expensive and/or harmful to the environment because of the use of hazardous chemical reagents.

To overcome this problem, an eco-friendly and cost-effective treatment consisting of the immersion of natural fibers in sodium bicarbonate solution was investigated in the last years by several researchers [32,43,44,45,46,47,48,49]. In particular, they showed that the interaction between the mildly alkaline solution and the fiber surface is considered similar to that of traditional alkalization treatment [43]. Even if the beneficial effect of sodium bicarbonate treatment on the mechanical properties of NFRPs was widely evidenced, no one has yet investigated how this new approach allows better retention of the dynamic mechanical stability of NFRPs under aggressive environments. 

Within this scope, the effectiveness of sodium bicarbonate treatment on the evolution of the dynamic mechanical properties of flax, jute and hybrid flax/jute fiber-reinforced composites under salt-fog environment was evaluated for the first time in the present paper. Epoxy composites reinforced with untreated (i.e., as received) and treated fibers were exposed to salt-fog environments and their damping properties were evaluated after 30 and 60 days of aging, respectively.

## 2. Materials and Methods 

In the present paper the evolution of the dynamic mechanical properties of flax, jute and hybrid flax/jute fiber-reinforced epoxy laminates [48] was analyzed by increasing their exposition time in a salt-fog environment. In particular, two kinds of balanced 2 × 2 twill weave woven fabrics were used as reinforcement, i.e., flax (areal weight 320 g/m^2^) and jute fabrics (400 g/m^2^), supplied by Lineo (Valliquerville, France) and Composites Evolution (Chesterfield, UK), respectively. SX8 EVO A epoxy resin, supplied by Mates Italiana s.r.l. (Segrate-Milano, Italy), mixed with SX8 EVO B hardener (100:30 mix ratio by weight) was used as a matrix. A vacuum-assisted resin infusion method was employed as a manufacturing method of composite panels, cured at 25 °C for 24 h and post-cured at 50 °C for 8 h. The untreated fabrics were dried at 40 °C for 24 h and at 103 °C for further 24 h to remove the moisture content. 

To pre-treat the natural reinforcement, flax and jute fabrics were soaked in a 10 wt.% distilled water sodium bicarbonate (i.e., NaHCO_3_) solution at 25 °C for 5 days, washed with distilled water and dried at 40 °C for 24 h and at 103 °C for further 24 h. All the compared laminates (see Table 1) showed fiber volume fraction equal to about 30%, while the voids content was lower than 5%, as reported in our previous paper [48]. In particular, the voids volume content ($v_{V}$) of each composite was evaluated by comparing its experimental and theoretical densities:

(1) $$v_{V}=\frac{\rho_{t}-\rho_{e}}{\rho_{e}},$$

where $\rho_{e}$ is the experimental density measured using a helium pycnometer model Pycnomatic ATC (Thermo Electron Corporation, Waltham, MA, US) and $\rho_{t}$ is the theoretical density, calculated with the following equation:

(2) $$\rho_{t}=\frac{1}{\left(\frac{W_{f}}{\rho_{f}}\right)+\left(\frac{W_{m}}{\rho_{m}}\right)},$$

where $\rho_{m}$ and $W_{m}$ are the density and the weight content of the epoxy matrix, whereas $\rho_{f}$ and $W_{f}$ are the density and the weight content of natural fiber, respectively.

Epoxy composite panels (25 cm × 25 cm) were exposed in a climatic chamber model SC/KWT 450 by Weiss (Germany) to salt-fog spray environment for up to 2 months, according to ASTM B 117 standard. After 30 and 60 days of salt-fog aging, specimens were cut through with a diamond saw from the center of each composite panel to perform the dynamic mechanical characterization. The evolution of the dynamic mechanical response of flax, jute and hybrid flax/jute laminates during aging exposition was done by comparing their mechanical properties with those of reference samples (i.e., unaged samples). The dynamic properties (i.e., storage modulus, loss modulus and tan δ) of each composite were evaluated through dynamic mechanical thermal tests (DMTAs), in accordance with ASTM D 4065 standard. The above characterization was carried out under three-point bending mode at constant frequency of 1 Hz, using a dynamic mechanical analyzer Metravib DMA+150 (Limonest, France). Three specimens (3 mm × 46 mm) per laminate were tested in nitrogen atmosphere from 25 °C to 140 °C at a heating rate of 3 °C/min. 

## 3. Results and Discussion

### 3.1. Effect of Treatment

**Figure 1:**
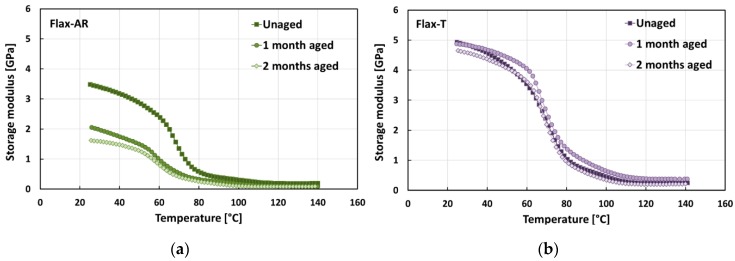
Typical storage modulus trends of flax composites in each aging condition: (**a**) Flax-AR composites; (**b**) Flax-T composites.

Figure 1 shows the typical storage modulus (E’) trends of flax fiber-reinforced composites as a function of their exposition time to salt-fog environment (i.e., 0 days or unaged, 30 days and 60 days). 

As is widely known [40,50], the storage modulus is typically associated with the material stiffness: it measures the material’s capability to store the energy applied. Two different regions of the storage modulus curve of fiber-reinforced polymers by varying temperature were evidenced, i.e., the first one below glass transition temperature (Tg) of the polymeric matrix and the second one above Tg, named glassy region and rubbery region, respectively. In the glassy region, the storage modulus of fiber-reinforced composite materials was high because the components (i.e., matrix and reinforcement) were extremely immobilized, allowing a stiff behavior. Vice versa, FRP components acquired mobility, losing their close packing arrangement in the rubbery region, thus leading to decrease of the storage modulus with temperature [40].

By comparing Figure 1a,b, it was noted that, in the unaged condition, sodium bicarbonate treatment allowed the composite stiffness to increase in the glassy region. In particular, the storage modulus E’ at 25 °C of Flax-T laminates (i.e., 5.0 ± 0.1 GPa) was about 47% higher than that of Flax-AR laminates (i.e., 3.4 ± 0.2 GPa). This result confirms that the adhesion between flax fibers and epoxy matrix becomes strongest due to the fiber treatment, as already stated in our previous papers [46,48]. 

It is worth noting that, during the exposition to the salt-fog environment, Flax-AR composites experienced noticeable decrements of E’ in the glassy region. In particular, laminates aged for 1 month and 2 months showed storage modulus values 32% and 49% lower than the reference (i.e., unaged laminates), respectively. Vice versa, a stable dynamic behavior during the exposition to the aggressive environment was evidenced by Flax-T composites. Indeed, the soaking of flax fabrics in NaHCO_3_ solution allowed to obtain a composite laminate able to better retain its storage dynamic modulus below glass transition temperature, i.e., in comparison to unaged samples the measured decrements of E’ at 25 °C were equal to just 3% and 8% after 1 month and 2 months of aging, respectively. This suggests that, in addition to the quasi-static mechanical stability [32], fiber treatment allows flax-reinforced epoxy composites also to better retain their dynamic behavior under salt-fog exposition.

Loss modulus E’’ represents the viscous response of the material (i.e., the material tendency to dissipate applied energy), whereas tan δ, also named damping or loss factor, is defined as the loss modulus to storage modulus ratio (E’’/E’). A high tan δ value is indicative of materials having a great non-elastic strain component while a low value indicates high elasticity. This means that, for fiber-reinforced composites, an increase of the fiber/matrix adhesion results in the reduction of tan δ due to the mobility decrement of the molecular chains surrounding the fiber/matrix interface [51,52]. As a consequence, the lower the energy loss of a composite system compared to its storage capacity, the greater the tan δ value [42]. 

**Figure 2:**
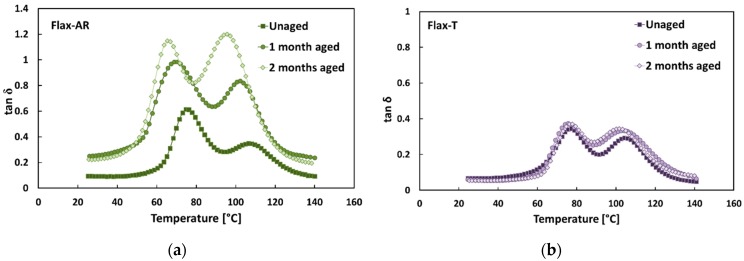
Typical tan δ trends of flax composites in each aging condition: (**a**) Flax-AR composites; (**b**) Flax-T composites.

Figure 2 shows the typical tan δ versus temperature trends of flax fiber-reinforced epoxy composites for each aging condition investigated. First of all, it is worth noting that two different tan δ peaks were evidenced for all samples (i.e., regardless the fiber treatment and aging exposition time). Several authors [38,53,54] stated that the first peak at lower temperatures is attributed to the glass transition temperature of the thermoset matrix, whereas the second one at higher temperatures is related to the micro-mechanical transition phenomena of the immobilized polymer layer between fiber and matrix, i.e., fiber–matrix interphase. 

The comparison of Figure 2a,b evidenced that, regardless the aging condition, Flax-AR laminates showed higher tan δ than those of Flax-T, thus confirming the beneficial effect of the sodium bicarbonate treatment on the fiber/matrix adhesion, even when flax fiber-reinforced composites were exposed to aggressive media such as salt-fog spray. 

**Figure 3:**
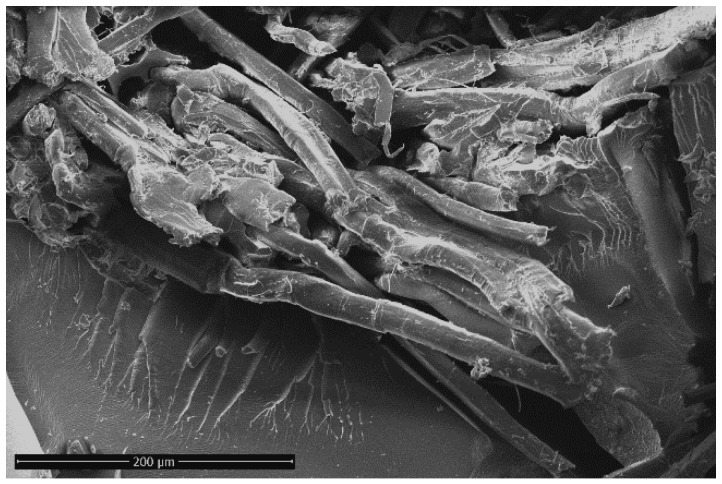
SEM micrograph of Flax-AR laminate surface after 60 days of salt-fog exposition.

Furthermore, Figure 2a shows that, after salt-fog aging, the tan δ peaks of Flax-AR laminates (i.e., 1-month and 2-month aged samples) shifted to lower temperatures and became wider than the unaged sample. This experimental evidence is mainly explained by coupling the softening of matrix, due to the composites’ exposition to the humid environment, and the low arrangement of the flax microfibrils network, as shown in Figure 3. FRP constituents gained local mobility, losing their packing arrangement in the rubbery region, thus favoring the shift toward lower temperature of tan δ [40].

From a morphological point of view, the Flax-AR laminate was characterized by low interfacial fiber–matrix adhesion, as can be seen from high tan δ peaks already shown in unaged conditions. This implies that preferential pathways for water diffusion can be identified in the interface between fibers and matrix, thus stimulating the triggering of premature water-induced degradation phenomena in the natural reinforced composite laminate. In particular, water weakens the physical bonds (i.e., van der Waals interaction) within the flax/matrix interface, as well as causing the hydrolysis of chemical bonds at the interface, thus leading to the occurrence of interfacial debonding phenomena [40]. As evidenced in Table 2, the temperature of both tan δ peaks decreased about 10 °C after 60 days of salt-fog exposition (i.e., from 75.4 °C to 65.5 °C, and from 105.6 °C to 95.2 °C, for the first and the second peak, respectively). As concerns the evolution of the peaks’ height, the first one varied from 0.57 to 1.15, whereas the second one varied from 0.32 to 1.13.

Vice versa, no noticeable variation was evidenced on the tan δ trends of Flax-T composites when varying the aging exposition time (Figure 2b). Both peaks remained slightly unchanged when varying the aging condition. In particular, after 60 days of salt-fog exposition, the temperature decrements were just equal to 0.7 °C (i.e., from 77.2 °C to 76.5 °C) and 2.4 °C (from 104.7 °C to 102.3 °C) for the first and the second peak, respectively (Table 2). At the same time, the height of the first peak varied from 0.37 to 0.38, whereas the second one varied from 0.28 to 0.37. These results are in good agreement with those achieved from the quasi-static flexural characterization [32], confirming the improved mechanical stability shown by Flax-T laminates in comparison to Flax-AR ones. 

It is noteworthy that the second peak showed a greater sensitivity to aging in the salt-fog environment than the first one. This indicates that the water diffusion phenomena, which are considered responsible for the aging activation phenomena [55], are activated initially at the interface between the fiber and the matrix and then evolve towards the bulk of the matrix, thus causing its softening. The Flax-T laminate, being characterized by an improved interfacial adhesion, strongly limited the water absorption by inhibiting or extending over time the subsequent aging effect on the epoxy matrix.

**Figure 4:**
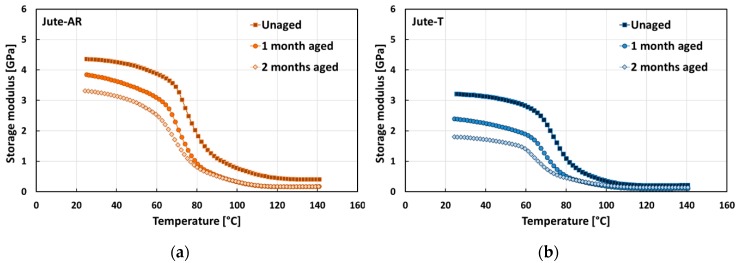
Typical storage modulus trends of jute composites in each aging condition: (**a**) Jute-AR composites; (**b**) Jute-T composites.

Figure 4 shows the typical storage modulus versus temperature trends of jute laminates for each aging condition investigated. Regarding unaged samples, it is worth noting that, unlike flax laminates, fiber treatment did not increase the composite stiffness in the glassy region. In particular, epoxy laminates reinforced with untreated or raw jute fabrics (i.e., Jute-AR) showed E’ at room temperature (i.e., 4.3 ± 0.1 GPa) about 47% higher than Jute-T laminates (i.e., 2.9 ± 0.3 GPa). This experimental result confirms that the adverse effect of the sodium bicarbonate treatment on the adhesion between jute fibers and epoxy resin leads to decreasing the dynamic modulus of the resulting composites [48].

As a consequence, Jute-T laminates experienced greater decrements of storage modulus in the glassy region due to the salt-fog exposition in comparison to their counterparts (i.e., Jute-AR laminates). In more detail, the storage modulus values at room temperature of Jute-AR-1m and Jute-AR-2m laminates were 10% and 18% lower than that of the unaged laminates, respectively. On the contrary, Jute-T composites evidenced decrements in the storage modulus at room temperature equal to 25% and 40%, after 1 month and 2 months of aging exposition, respectively.

**Figure 5:**
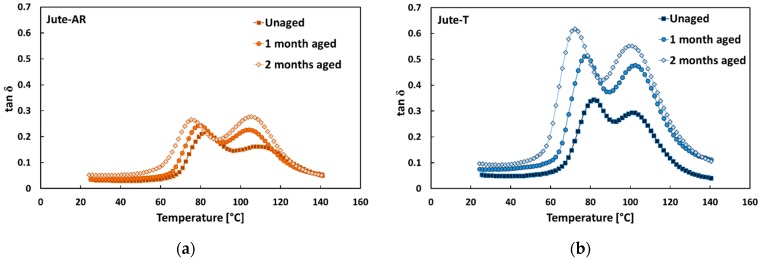
Typical tan δ trends of jute composites in each aging condition: (**a**) Jute-AR composites; (**b**) Jute-T composites.

By observing the typical tan δ versus temperature trends of jute composites at varying aging conditions (Figure 5), it is evidenced that, regardless of fiber treatment, both tan δ peaks became higher, also shifting to lower temperatures, when increasing the salt-fog exposition time. Since the damping behavior of fiber-reinforced composites is greatly influenced by the shear stress concentrations at the fiber–matrix interfaces, as well as by the viscoelastic energy dissipation within the polymeric matrix [51], these results are attributed both to the matrix softening and to the fiber–matrix interface degradation phenomena, due to the composites’ exposition to salt-fog. As already stated, no beneficial effect on fiber–matrix compatibility was achieved after jute fiber treatment. Consequently, Figure 5 clearly shows that, regardless the aging time exposition, tan δ values of Jute-AR laminates were lower than those of Jute-T in the whole temperature range because they were characterized by higher mobility of the molecular chains both in the matrix bulk and surrounding the fiber/matrix interface. 

This result indicates that composites reinforced with untreated Jute fibers (i.e., Jute-AR) are characterized by better fiber–matrix interfacial adhesion than surface treated ones (i.e., Jute-T) [47]. Due to the soaking of jute fibers in the NaHCO_3_ solution, a reduction of lignin and hemicellulose components from the fiber bulk occurred [44], thus favoring the triggering of damage phenomena at the interface. Indeed, Jute-T laminates showed a larger decrement in their dynamic mechanical performances than Jute-AR laminates during the aging exposition, due to their higher sensitivity to a water-based environment.

In detail, it was found that Jute-T laminates showed greater variations of both tan δ peaks with increasing exposition time to salt-fog environment. In particular, after 60 days in the climatic chamber, the temperature of the first peak decreased from 82.0 °C to 76.3 °C (i.e., ΔT = 5.8 °C) and from 81.5 °C to 72.0 °C (i.e., ΔT = 9.5 °C) for Jute-AR and Jute-T laminates, respectively (Table 3). With regard to the second tan δ peak, its temperature decrements were found equal to 3.3 °C (i.e., from 108.3 °C to 105.0 °C) and 4.1 °C (i.e., from 109.0 °C to 104.9 °C) for Jute-AR and Jute-T laminates, respectively. At the same time, Jute-T laminates showed increments from 0.33 to 0.62 and from 0.23 to 0.51 in the heights of the first and second tan δ peaks, respectively. Vice versa, epoxy laminates reinforced with jute untreated fibers (i.e., Jute-AR) showed lower height increments of both peaks (i.e., from 0.22 to 0.31 for the first peak and from 0.16 to 0.24 for the second one). These results are ascribed to the weaker fiber–matrix interfacial adhesion evidenced by Jute-T laminates in comparison to Jute-AR ones [32,33], which triggers water diffusion and, consequently, the composite aging after a short time of exposition to salt-fog environment.

### 3.2. Effect of Stacking Sequence

**Figure 6:**
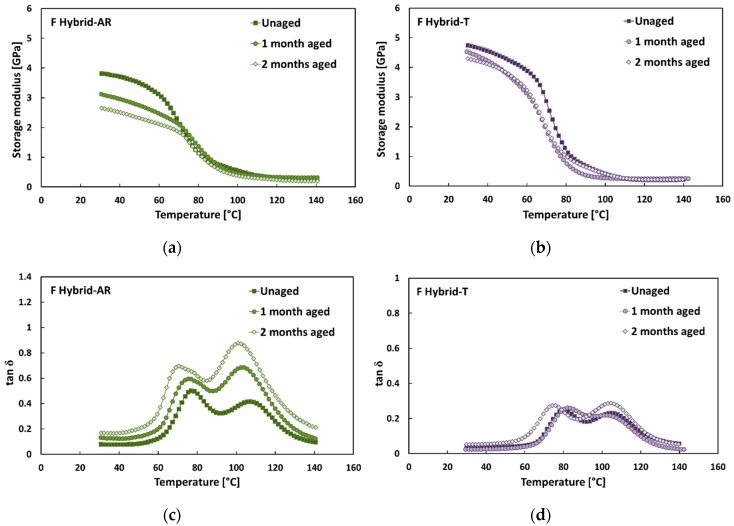
(**a**,**b**) Typical storage modulus and (**c**,**d**) tan δ trends of F-Hybrid composites in each aging condition.

The typical trends of the damping properties (i.e., storage modulus E’ and tan δ) versus temperature of F Hybrid laminates are shown in Figure 6 for each aging condition. By observing this figure, it can be noted that, even for hybrid stacking sequence, NaHCO_3_ treatment allowed to modify the dynamic properties of the resulting composites. As shown in Figure 6a,b, F Hybrid-T laminates evidenced higher stiffness in the glassy region than their counterparts (i.e., F Hybrid-AR), regardless the aging time exposition. In particular, the average storage moduli E’ measured at 25 °C of laminates reinforced with treated fibers (i.e., F Hybrid-T) were about 22%, 44% and 64% higher than those of F Hybrid-AR, after 0, 30 and 60 days in salt-fog environment, respectively. 

Accordingly, the comparison of Figure 6c,d evidenced that F Hybrid-AR unaged laminates showed higher tan δ values than Flax-T unaged ones, in the whole temperature range. In addition, the exposition to salt-fog clearly modified the tan δ curves of F Hybrid-AR laminates, since both tan δ peaks became higher and shifted to lower temperatures when increasing the exposition time. After 60 days of salt-fog exposition, the tan δ peak temperatures decreased about 8 °C (i.e., from 75.7 °C to 68.0 °C) and 5 °C (i.e., from 107.0 °C to 101.9 °C), for the first and the second one, respectively. As regards the peaks’ heights, the first peak grew from 0.48 to 0.69, whereas the second one grew from 0.42 to 0.87 (Table 4).

Vice versa, the typical tan δ trends of F Hybrid-T composites remained almost unchanged when varying the exposition time to salt-fog environment, as shown in Figure 6d, i.e., no noticeable variation was observed by comparing tan δ peaks of unaged and salt-fog aged F Hybrid-T laminates.

Both peaks remained unchanged when varying the aging condition. In particular, after 60 days of salt-fog exposition, the temperature decrements for both peaks were just equal to 1.8 °C (i.e., from 79.4 °C to 77.6 °C and from 104.3 °C to 102.5 °C for the first and the second one, respectively). At the same time, the height of the first peak varied from 0.24 to 0.28, whereas the second one varied from 0.20 to 0.28. 

All these results can be ascribed to the different interactions of the two natural fibers with the sodium bicarbonate treatment. If, on one hand, the bicarbonate treatment was widely proven beneficial to the adhesion between flax fibers and epoxy matrix, on the other hand, it was equally evident that the same treatment weakened the jute fiber–epoxy matrix interface. Consequently, it is reasonable to consider that F Hybrid-T laminates showed better damping properties in comparison to their untreated counterparts (i.e., F Hybrid-AR) because the stacking sequence of F Hybrid laminates is characterized by more flax-reinforced laminae than jute ones (i.e., three versus two). Moreover, these hybrid laminates present flax-reinforced laminae as external layers. The bending performances of the composite laminates are heavily influenced by these external laminae that suffer the higher longitudinal stresses, unlike the central laminae where the neutral axis is located. Furthermore, it can be evidenced that F Hybrid-T laminates showed better damping properties in comparison to their untreated counterparts (i.e., F Hybrid-AR) because the stacking sequence of F Hybrid laminates is characterized by a larger amount of flax-reinforced laminae than jute ones (i.e., three reinforced laminae versus two).

On the contrary, J Hybrid laminates have three epoxy-based laminae reinforced with jute fibers versus two reinforced with flax ones in their stacking sequence. In addition, the external laminae supporting the highest longitudinal stresses are reinforced with jute fibers. Consequently, the detrimental effect of the treatment on jute fiber–epoxy matrix adhesion, in addition to worsening the damping properties of J Hybrid-T laminates in comparison to the laminates reinforced with as received fabrics (i.e., J Hybrid-AR) in the unaged condition [32], reduced their service life when exposed to salt-fog. 

**Figure 7:**
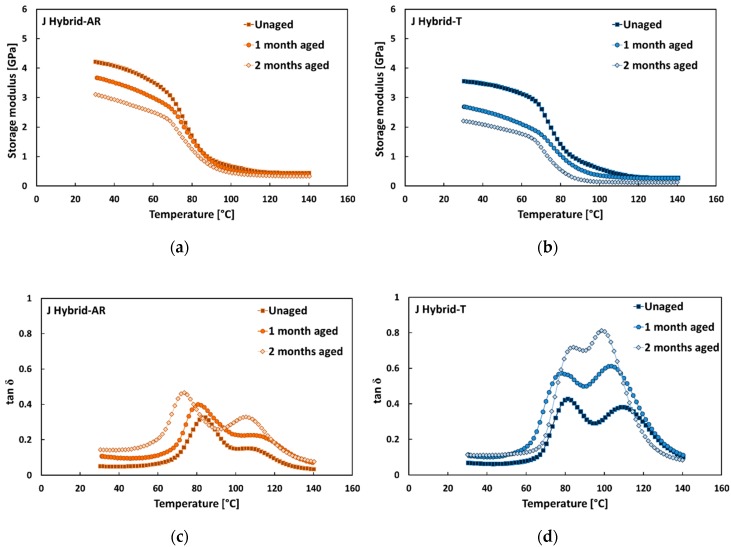
(**a**,**b**) Typical storage modulus and (**c**,**d**) tan δ trends of J-Hybrid composites in each aging condition.

Indeed, J Hybrid-T laminates showed the greater decrement of the storage modulus E’ in the glassy region with the exposition time to salt-fog in comparison to J Hybrid-AR laminates, as evidenced by comparing Figure 7a,b. In particular, it was found that E’ measured at room temperature of J Hybrid-T laminates after 1 month and 2 months under salt-fog environment was 23% and 37% lower than unaged laminates, respectively. On the other hand, J Hybrid-AR composites better retained their dynamic stiffness, showing a reduction of the storage modulus at room temperature equal to 12% and 24% after 1 month and 2 months of aging exposition, respectively.

Concerning the effect of salt-fog exposition on tan δ trends, Figure 7c,d evidences that both tan δ peaks became higher and shifted to lower temperatures when increasing the salt-fog exposition time, for laminates reinforced with treated or as received fabrics. However, it is worth noting that tan δ values of J Hybrid-AR laminates were lower than those of J Hybrid-T in the whole temperature range, regardless the aging exposition time. In accordance with the evolution of the storage modulus, hybrid laminates reinforced with treated fabrics (i.e., J Hybrid-T) showed greater variations of both tan δ peaks with salt-fog exposition time in comparison to J Hybrid-AR laminates. Table 5 evidences that the temperature of the first peak slightly decreased 5.4 °C (i.e., from 79.9 °C to 74.5 °C) and 10.1 °C (i.e., from 81.9 °C to 71.8 °C) for J Hybrid-T and J Hybrid-AR laminates after 60 days under salt-fog, respectively. The second tan δ peak decreased from 109.6 °C to 99.9 °C (i.e., ΔT = 9.7 °C) and 106.8 °C to 103.6 °C (i.e., ΔT = 3.2 °C) for J Hybrid-T laminates and J Hybrid-AR, respectively.

At the same time, J Hybrid-T laminates increased the heights of first and second tan δ peaks from 0.43 to 0.63 and from 0.38 to 0.86 after 60 aging days, respectively. Vice versa, hybrid laminates reinforced with untreated fabrics (i.e., J Hybrid-AR) showed reduced height increments of both peaks (i.e., from 0.33 to 0.46 for the first peak and from 0.18 to 0.33 for the second one). This phenomenon can be related to the effect induced by the sodium bicarbonate treatment that, as reported in [48], leads to a significant enhancement of the quasi-static performances of flax-reinforced composites and, conversely, a reduction in strength and stiffness of jute-based composites.

### 3.3. Damping Properties Versus Moisture Content

Useful information can be extrapolated by analyzing the correlation between the damping properties variation and the progressive water absorption evidenced by the NFRPs during their exposition to the salt-fog condition. This approach allows to discriminate how mechanical parameters are more affected by degradative phenomena. In such a context, Table 6 reports the water uptake values evidenced by all the compared laminates after 30 days and 60 days under salt-fog condition, respectively.

It is worth noting that all samples evidenced a relevant hydrophilic behavior, showing weight gain values in the range 10–13% after 30 days of salt-fog exposition. Moreover, it was found that Flax-AR and Jute-T laminates were the most sensitive laminates to wet environments. This indicates that the water uptake is not related to the water sorption capability of natural fibers or epoxy matrix, but it is mainly related to competing fiber–matrix phenomena that synergistically play an important role in the degradative process of these NFRP materials. 

**Figure 8:**
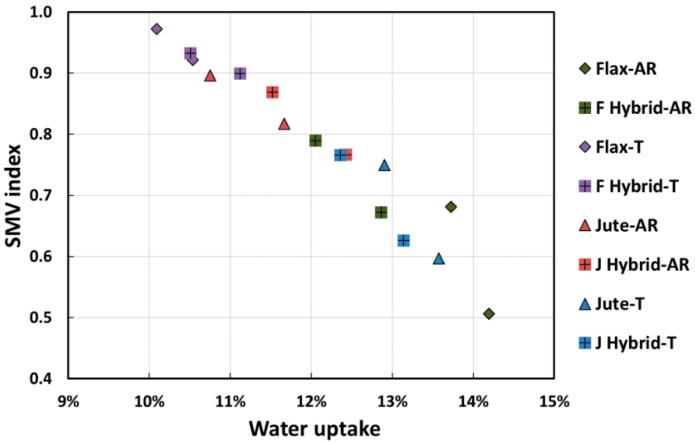
Relationship between moisture content and the variation of storage modulus E’ measured at room temperature.

**Figure 9:**
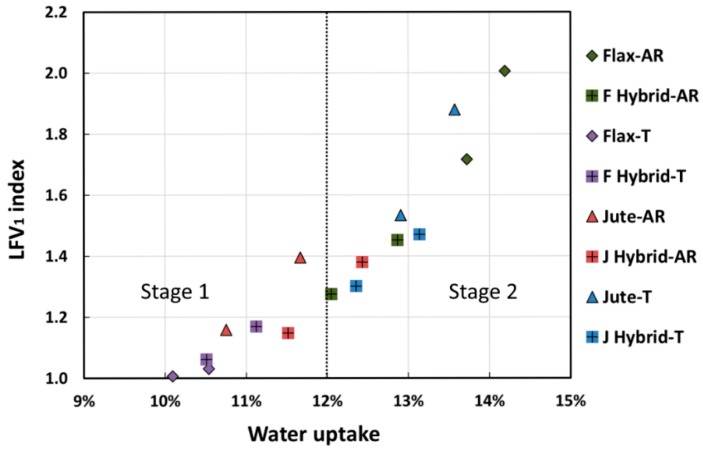
Relationship between moisture content and the variation of first loss factor peak (LFV_1_ index).

**Figure 10:**
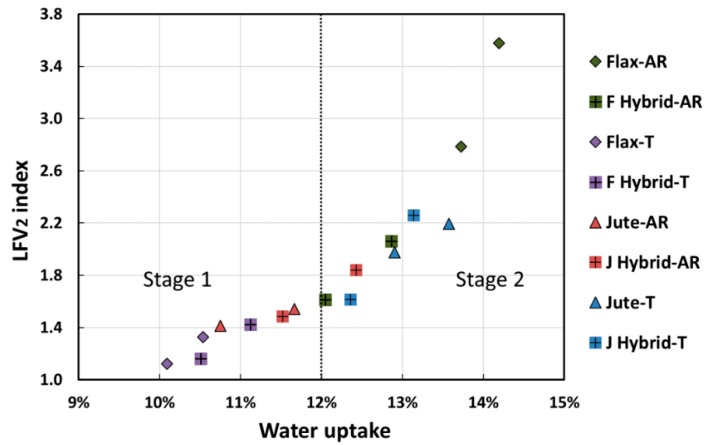
Relationship between moisture content and the variation of second loss factor peak (LFV_2_ index).

In order to relate the dynamic mechanical performance modification with environmental aging in wet conditions, the relationships between the variation of damping properties of all the resulting laminates and their moisture content are shown in Figure 8, Figure 9 and Figure 10. In particular, the variation of each damping property was calculated as follows:

(3) $$∆P=P_{t}/P_{0},$$

where *P_t_* is the value of the damping property of the laminate under salt-fog condition for a specific time *t* (i.e., 1 or 2 months) and *P*_0_ is the value of the same property at the beginning of the aging campaign (i.e., for the unaged condition).

In particular, Figure 8 shows that moisture had a significant influence on the storage modulus E’ measured at 25 °C of NFRP laminates. It was clearly found a linear relationship between the storage modulus variation (indicated as SMV index) and the water uptake. Similar to what was observed by Wang and Petru [40], a clear decrease of the dynamic stiffness was found with increasing the composites’ moisture content. Due to the polysaccharide content of natural fibers and the voids and cracks in the epoxy matrix bulk, flax and jute fibers tended to absorb a great amount of water when composites were exposed to humid environments such as a marine one [10,23]. Hence, the penetration of water molecules and ions (i.e., Na^+^ and Cl^−^) within the composite structure led to softening both matrix and natural reinforcing fibers, as well as chemically and physically degrading the inherently weak fiber/matrix interface. 

Furthermore, it is worth noting that composites having the strongest fiber–matrix adhesion (i.e., Flax-T and F Hybrid-T laminates) [48] were characterized by lower moisture absorptions and, consequently, more contained stiffness reductions (as visible by the positioning of purple dots in the graph of Figure 8). On the contrary, the highest values of moisture absorption and storage modulus reductions were shown by composites with weak fiber–matrix adhesion (i.e., Flax-AR, Jute-T, J Hybrid-T, F Hybrid-AR). These results can be explained, taking into account the different interactions of the investigated natural fibers (i.e., flax and jute) with the sodium bicarbonate treatment. As shown in our previous paper [48], bicarbonate treatment softens jute fibers, due to their higher amount of lignin (i.e., up to 25–27%) in comparison to flax fibers (i.e., up to 5%) [1,56,57]. Conversely, this mildly alkaline treatment is able to remove the impurities from the surface of flax fibers without any chemical interaction with the fiber bulk because of the lower lignin content and the more compact structure, compared to jute fibers [58]. These different interactions allow composites reinforced with treated flax fibers to have a stronger fiber–matrix adhesion in comparison to those reinforced with raw fibers. Conversely, a slight worsening of adhesion between jute fibers and epoxy resin is achieved after sodium bicarbonate treatment.

In accordance with the trend of the dynamic storage modulus, the height of loss factor (i.e., tan δ) peaks increased with the moisture content, as shown in Figure 9 and Figure 10. 

It is well known that the loss factor versus temperature trend of FRP materials is influenced by the incorporation of fibers in the polymeric matrix, due to the energy dissipation in the matrix bulk and to the shear stress concentrations at the fiber–matrix interfaces [51]. In particular, composites with a weak fiber–matrix interface show high and large loss factor peaks because they tend to dissipate more energy in comparison to FRPs having the strongest fiber–matrix adhesion [54,59]. Moreover, when composites are exposed to a humid environment, the value of loss factor peaks increases with the exposition time to humid environments and, consequently, moisture content [40]. This detrimental effect is more pronounced for composites characterized by a weak fiber–matrix interface because they dramatically suffer mechanical performance degradation when exposed to humid environments during their service life [12,13].

By observing the trend of LFV_1_ (i.e., loss factor variation for the first peak) index as a function of the moisture content, it was possible to identify two different stages of the degradative process (Figure 9). The first one (i.e., Stage 1) occurring at low water uptake values, was characterized by limited variation of the loss factor for the first peak. This could be ascribed to a water uptake not relevant enough to modify the mobility of the polymer chains in the matrix bulk. On the other hand, when the water absorption phenomenon became significant, a relevant enhancement of LFV_1_ index was highlighted, as evidenced by the increased slope of the curve trend (i.e., Stage 2). Water molecules are able to diffuse inside the laminate structure, thus triggering softening phenomena of the matrix with, as a consequence, greater LFV_1_ index values occurring. Indeed, samples evidencing the highest water absorption showed LFV_1_ index values higher than 1.5.

As evidenced in Figure 9, the water uptake level equal to 12% can be considered as a threshold transition value between the two abovementioned stages for these natural composite laminates.

Similar considerations were drawn by observing the LFV_2_ (i.e., loss factor variation for the second peak) index versus water uptake trend shown in Figure 10. It was of upmost importance to evidence that the second peak variation was more affected by water uptake in comparison to the first peak. Even in this case, two different degradative stages were identified at low and high water uptake values, respectively. Noticeable variations of the second peak height were evidenced already in the first stage, i.e., LFV_2_ values equal to about 1.4 for water uptake lower than 12%. These experimental results confirmed that the fiber–matrix adhesion weakening was strongly correlated with the water absorption experienced by the laminate in the beginning phase of the salt-fog exposition.

Therefore, this stage can be considered a discriminating factor concerning the micro-mechanical transition phenomena of the immobilized polymer layer between fiber and matrix (i.e., fiber–matrix interphase). 

On this basis, it is reasonable to consider that the water diffusion inside the composite structure mainly depended on a weak fiber–matrix adhesion that, in turn, represented the main factor activating degradative phenomena. The transition between the two degradative stages was identified, also in this case, at water uptake values equal to 12%. Beyond this threshold limit, the LFV_2_ index became higher than 2 (i.e., up to 3.5 for Flax-AR samples, for instance), thus indicating a serious loss of the adhesion between the main components of the composite (i.e., fiber and matrix).

Due to the above reasons, composites having the weakest fiber–matrix adhesion, such as Flax-AR and Jute-T, evidenced the highest increments in loss factor peaks as well as the greatest moisture absorption during their exposition to the salt-fog environment. On the contrary, the beneficial effect of the NaHCO_3_ treatment on the adhesion between flax fibers and epoxy resin allowed composites such as Flax-T and F-Hybrid T to reduce their moisture absorption and, as a consequence, to better retain their damping peaks. Similarly, composites with inherently good fiber–matrix adhesion, such as Jute-AR and J Hybrid-AR, showed low moisture adsorption and slight variations in both loss factor peaks, also after long exposition time in the salt-fog environment.

**Figure 11:**
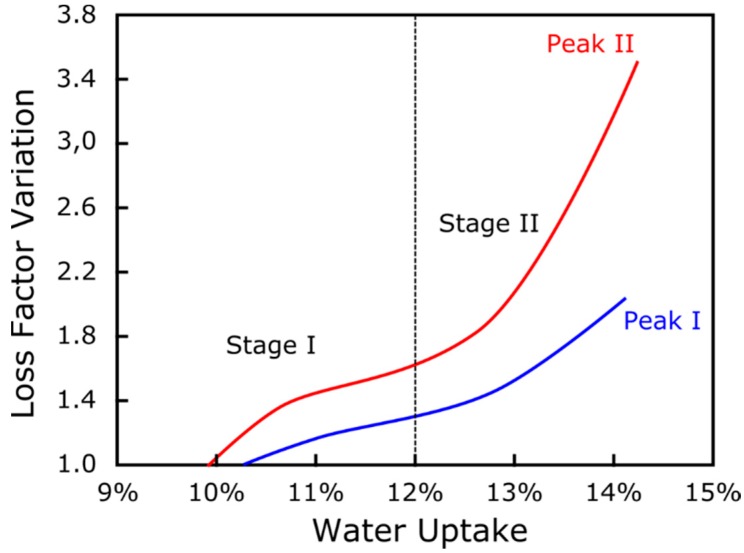
Scheme of LFV_1_ and LFV_2_ index versus water uptake trends comparison.

With the aim to better correlate the degradative phenomena evolution, a comparison of LFV_1_ and LFV_2_ versus water uptake trends is schemed in Figure 11. Concerning Stage 1, LFV_2_ was equal to about 1.4, thus indicating peak variation of about 40%, twice the variation of the first peak. In addition, the slope of the LFV_2_ trend was noticeably higher than that evidenced for LFV_1_. These experimental results further confirmed that the main limiting factor in the application of NFRPs in humid or wet conditions is represented by the worsening effect on the fiber–matrix adhesion of these environments. This degradation phenomenon is much more sensitive than other ones such as matrix softening, which, although giving an appreciable contribution on aging degradation, can be considered, in this case, a sub-degradation step of the global degradation mechanisms.

Further studies will be aimed to better discriminate among the different damage evolution stages occurring in critical environmental conditions, such as salt-fog spray. This will provide added value in the identification of synergistic actions between various degradative mechanisms that contribute to the degradation of the laminate. However, the achieved results are promising and potentially suitable to integrate the design knowledge of natural composite laminates and their durability performance under specific environmental conditions during the design phase.

## 4. Conclusions

Since humid environments can be considered as the most critical factors reducing the service life of natural fiber-reinforced composites, the present paper aims to deeper understand how the exposition to aggressive environments such as salt-fog spray conditions can influence the dynamic mechanical response of these materials. 

Within this scope, the damping properties of flax, jute and hybrid flax/jute epoxy based composites were analyzed as a function of their exposition time to salt-fog. Furthermore, it was investigated how sodium bicarbonate treatment can allow these composites to better retain their dynamic mechanical response under aging conditions. 

By way of summarizing, it can be drawn from the experimental campaign that:

− All the NFRP materials (i.e., regardless the stacking sequence) experienced decreases of the storage modulus and glass transition temperature, as well as the increase of the loss factor peaks with salt-fog exposition time and moisture content;
− Full laminates reinforced with flax fibers or hybrid laminates having more flax fabrics than jute ones in their stacking sequence evidenced a clear effectiveness of NaHCO_3_ treatment on the retention of their damping properties during exposition to salt-fog environments;
− Conversely, the proposed treatment had no beneficial effect on jute-based laminates (in terms of preservation of damping properties), thus evidencing that the efficacy of treatment is strictly related to the fiber’s chemical composition;
− A relationship between the decrease of the damping properties of the composites and their progressive increase of water absorption during the salt-fog exposition was evaluated in order to better discriminate how mechanical parameters are affected by degradative phenomena, thus indicating that the fiber–matrix interfacial water diffusion plays a relevant role in the degradation triggering and propagation.

## Figures and Tables

**Table 1:** List of composite laminates.

Code	Stacking Sequence ^1^	Fabrics
Flax-AR	[F]_5_	as received
Flax-T	[F]_5_	treated
Jute-AR	[J]_5_	as received
Jute-T	[J]_5_	treated
F Hybrid-AR	[F/J/F/J/F]	as received
F Hybrid-T	[F/J/F/J/F]	treated
J Hybrid-AR	[J/F/J/F/J]	as received
J Hybrid-T	[J/F/J/F/J]	treated

^1^ F = twill weave flax fabric; J = twill weave woven jute fabric.

**Table 2:** Damping properties of flax composites for each aging condition.

Aging Days	Flax-AR	Flax-T	Flax-AR	Flax-T
	**First Peak T [°C]**		**First Peak Height**	
0	75.4 ± 1.5	77.2 ± 0.5	0.57 ± 0.04	0.37 ± 0.03
30	70.2 ± 0.4	76.2 ± 0.7	0.99 ± 0.01	0.37 ± 0.00
60	65.5 ± 0.5	76.5 ± 1.0	1.15 ± 0.01	0.38 ± 0.02
	**Second Peak T [°C]**		**Second Peak Height**	
0	105.6 ± 1.3	104.7 ± 1.3	0.32 ± 0.02	0.28 ± 0.01
30	102.6 ± 2.7	105.3 ± 1.6	0.88 ± 0.05	0.31 ± 0.02
60	95.2 ± 1.3	102.3 ± 2.3	1.13 ± 0.12	0.37 ± 0.05

**Table 3:** Damping properties of jute composites for each aging condition.

Aging Days	Jute-AR	Jute-T	Jute-AR	Jute-T
	First Peak T [°C]		First Peak Height	
0	82.0 ± 0.1	81.5 ± 0.7	0.22 ± 0.01	0.33 ± 0.01
30	78.6 ± 0.6	76.2 ± 0.8	0.26 ± 0.02	0.51 ± 0.00
60	76.3 ± 1.1	72.0 ± 1.6	0.31 ± 0.05	0.62 ± 0.01
	**Second Peak T [°C]**		**Second Peak Height**	
0	108.3 ± 1.0	109.0 ± 4.6	0.16 ± 0.01	0.23 ± 0.07
30	106.7 ± 2.1	108.0 ± 2.4	0.22 ± 0.01	0.46 ± 0.05
60	105.0 ± 1.9	104.9 ± 2.5	0.24 ± 0.04	0.51 ± 0.08

**Table 4:** Damping properties of F Hybrid composites for each aging condition.

Aging Days	F Hybrid-AR	F Hybrid-T	F Hybrid-AR	F Hybrid-T
	First Peak T [°C]		First Peak height	
0	75.7 ± 0.3	79.4 ± 0.8	0.48 ± 0.03	0.24 ± 0.01
30	73.0 ± 1.5	81.3 ± 0.6	0.61 ± 0.04	0.25 ± 0.01
60	68.0 ± 0.9	77.6 ± 3.5	0.69 ± 0.01	0.28 ± 0.02
	**Second Peak T [°C]**		**Second Peak Height**	
0	107.0 ± 0.7	102.2 ± 2.4	0.42 ± 0.04	0.20 ± 0.03
30	102.2 ± 2.4	99.9 ± 1.4	0.68 ± 0.02	0.23 ± 0.01
60	101.9 ± 2.9	102.5 ± 4.7	0.87 ± 0.01	0.28 ± 0.01

**Table 5:** Damping properties of J Hybrid composites for each aging condition.

Aging Days	J Hybrid-AR	J Hybrid-T	J Hybrid-AR	J Hybrid-T
	First Peak T [°C]		First Peak Height	
0	81.9 ± 0.9	79.9 ± 0.2	0.33 ± 0.02	0.43 ± 0.00
30	80.5 ± 2.0	80.5 ± 3.4	0.38 ± 0.02	0.56 ± 0.12
60	71.8 ± 0.1	74.5 ± 5.5	0.46 ± 0.02	0.63 ± 0.04
	**Second Peak T [°C]**		**Second Peak Height**	
0	106.8 ± 1.1	109.6 ± 1.1	0.18 ± 0.03	0.38 ± 0.04
30	105.9 ± 5.0	100.1 ± 3.0	0.27 ± 0.07	0.62 ± 0.01
60	103.6 ± 4.2	99.9 ± 2.7	0.33 ± 0.02	0.86 ± 0.09

**Table 6:** Water absorption percentage of all laminates in each aging condition.

Laminate	30 Days	60 Days
Flax-AR	13.7 ± 0.5	14.2 ± 0.5
Flax-T	10.1 ± 0.5	10.5 ± 0.5
Jute-AR	10.7 ± 0.3	11.7 ± 0.6
Jute-T	12.9 ± 0.3	13.6 ± 0.7
F Hybrid-AR	12.1 ± 0.7	12.9 ± 0.5
F Hybrid-T	10.5 ± 0.5	11.1 ± 0.5
J Hybrid-AR	11.5 ± 0.6	12.4 ± 0.5
J Hybrid-T	12.4 ± 0.3	13.1 ± 0.7
